# Potential Antifungal Targets for *Aspergillus* sp. from the Calcineurin and Heat Shock Protein Pathways

**DOI:** 10.3390/ijms232012543

**Published:** 2022-10-19

**Authors:** Robert Ancuceanu, Marilena Viorica Hovaneț, Maria Cojocaru-Toma, Adriana-Iuliana Anghel, Mihaela Dinu

**Affiliations:** 1 Faculty of Pharmacy, Carol Davila University of Medicine and Pharmacy, 020956 Bucharest, Romania; 2 Faculty of Pharmacy, Nicolae Testemițanu State University of Medicine and Pharmacy, 2025 Chisinau, Moldova

**Keywords:** *Aspergillus*, antifungal, calcineurin, heat shock proteins, signaling pathways, CrzA, rcnA, pmcC, brlA

## Abstract

*Aspergillus* species, especially *A. fumigatus*, and to a lesser extent others (*A. flavus, A. niger, A. terreus*), although rarely pathogenic to healthy humans, can be very aggressive to immunocompromised patients (they are opportunistic pathogens). Although survival rates for such infections have improved in recent decades following the introduction of azole derivatives, they remain a clinical challenge. The fact that current antifungals act as fungistatic rather than fungicide, that they have limited safety, and that resistance is becoming increasingly common make the need for new, more effective, and safer therapies to become more acute. Over the last decades, knowledge about the molecular biology of *A. fumigatus* and other *Aspergillus* species, and particularly of calcineurin, Hsp90, and their signaling pathway proteins, has progressed remarkably. Although calcineurin has attracted much interest, its adverse effects, particularly its immunosuppressive effects, make it less attractive than it might at first appear. The situation is not very different for Hsp90. Other proteins from their signaling pathways, such as protein kinases phosphorylating the four SPRR serine residues, CrzA, rcnA, pmcA-pmcC (particularly pmcC), rfeF, BAR adapter protein(s), the phkB histidine kinase, sskB MAP kinase kinase, zfpA, htfA, ctfA, SwoH (nucleoside diphosphate kinase), CchA, MidA, FKBP12, the K27 lysine position from Hsp90, PkcA, MpkA, RlmA, brlA, abaA, wetA, other heat shock proteins (Hsp70, Hsp40, Hsp12) currently appear promising and deserve further investigation as potential targets for antifungal drug development.

## 1. Introduction

Species belonging to the genus *Aspergillus* are filamentous fungi, widespread in various environments, where they live as saprophyte organisms [1]. Their spores are ubiquitous in the atmosphere and have tiny sizes (less than four μm) that allow them to be easily inhaled into the lower respiratory tract [2]. Although the known number of *Aspergillus* species exceeds 250, only a handful are known to be associated with human infections, mostly *A. fumigatus* Fresenius (50–60% of all aspergillosis cases) and, to a lesser extent, *A. flavus* Link, *A. niger* van Tieghem, and *A. terreus* Thom (each responsible for 10–15% of all aspergillosis cases) [2]. Although usually less frequently encountered, other *Aspergillus* species can also be problematic for clinical practice: *A. ustus* (Bainier) Thom & Church and *A. lentulus* Balajee & K.A. Marr are increasingly identified as agents responsible for invasive aspergillosis and are resistant to amphotericin B and at least some of the azoles. *A. terreus* also manifests intrinsic resistance to amphotericin B [3,4].

## 2. Aspergillosis

As for the large majority of fungi, *Aspergillus* species are rarely pathogenic for humans with a healthy immune system, but they may be very nosogenic for immunocompromised patients, i.e., they are opportunistic pathogens [5]. Such immunocompromised patients are those undergoing an organ transplant [5], those with neutropenia induced by antitumor chemotherapy, or under corticosteroid treatment [6]. Patients with impaired NADPH oxidase complexes, STAT3 or CARD9 signaling pathways, and those with leukocyte adhesion deficiencies or severe congenital neutropenia, are also susceptible to fungal infections, particularly aspergillosis [7]. Lung transplant patients, especially, are vulnerable to infections caused by *Aspergillus* species, and such conditions are associated with a high rate mortality rate [8]. In tropical and subtropical zones, *Aspergillus* and other filamentous fungi are the most frequent cause of fungal keratitis (unlike the temperate climates, where *Candida* sp. represents the leading cause) [9]. 

Until lately, finding *Aspergillus* in respiratory biological specimens was typically disregarded as a contaminant, except for cases where the patient was considered immunocompromised [10]. Because it is now accepted that severe disease states (e.g., sepsis) can have a strong negative effect on immunity, and assessing the extent of immunosuppression is challenging in ICU patients, evidence of aspergillosis is currently to be taken seriously in such patients [10]. 

Chronic pulmonary aspergillosis is a disease that in Europe only affects almost a quarter million persons; unlike invasive aspergillosis, these patients are not immunocompromised [11]. *A. fumigatus* is often found in respiratory secretions of patients with cystic fibrosis (both children and adults); this does not indicate harmless colonization, although the pathophysiology and clinical management have not been clarified [12]. It tends to be acknowledged, however, that *Aspergillus* colonization is likely to worsen the evolution of cystic fibrosis even in the absence of allergic bronchopulmonary aspergillosis [13]. A retrospective cohort study in cystic fibrosis patients found that for over a decade (from 1997 to 2007), the frequency of filamentous fungi isolates (mostly *Aspergillus* species) increased from only 2% to about 28.7% [14]. Moreover, the presence of *A. fumigatus* in biological samples of patients with chronic respiratory diseases is associated with more frequent manifestations of symptoms, lung fibrosis, and diabetes mellitus [15]. It has been estimated that *Aspergillus* species cause over 200,000 cases of invasive aspergillosis each year. Over 1.2 million patients are affected by chronic pulmonary aspergillosis (CPA), and between 5 and 10 million patients suffer from allergic bronchopulmonary aspergillosis (ABPA) and severe asthma with fungal sensitization (SAFS) [16]. These are only imperfect estimates because “epidemiological data for fungal infections are notoriously poor” due to frequent misdiagnosis and lack of active data collection on their impact (in the United States, except for coccidioidomycosis, no other fungal disease must be reported to the CDC) [17]. 

Costs associated with *Aspergillus* infections are relatively high; the estimates (per case) vary depending on costs considered and assumptions, from EUR 8351–11,821 (when only incremental hospitalization and antifungal product costs are considered) to EUR 26,596–83,300 (when all direct costs are included) [18]. In the USA, it has been estimated that *Aspergillus* infections are responsible for about USD 1.2 billion (14,820 cases) per year [19]. 

Both reliable diagnosis and successful treatment of aspergillosis remain a challenge; the presence of the species in a sample is not sufficient to confirm the infection [20], and the therapy is “limited to only a handful of antifungal agents” [21]. Invasive aspergillosis is associated with a gloomy prognosis: its all-cause mortality is estimated to be about 40% at 12 weeks [22]. A review paper from 2018 reported a case fatality rate of 29% in patients with hematological malignancies (almost one in every three patients), which was considered relatively low in comparison with an 88% case fatality rate estimated in 2001 [23]. The survival rates used to be much worse, but the clinical availability of azoles (itraconazole, voriconazole, posaconazole, and, more recently, isavuconazole) resulted in improvements that have been described as “dramatical” [24]. However, antifungal resistance to azoles has emerged and is likely to grow and evolve; the available data suggest that its impact on the clinical outcome is gloomy [25]. The clinical use of polyenes (the primary representative being amphotericin B) is limited by their toxicity [26]. The efficacy of the current antifungals is also restrained by the fact that they act as fungistatic rather than as fungicidal agents, arresting fungal growth but not eradicating the fungal cells [27]. 

About 16 years ago, the Antimicrobial Availability Task Force of the Infectious Diseases Society of America, discussing aspergillosis, concluded that “more-efficacious and better-tolerated therapies are needed. Orally available compounds would be highly useful” [28]. These have remained mostly desiderata because little progress has been made in this respect, and these needs are as relevant today as they were when first expressed. 

Because human and fungal cells are both eukaryotes, the latter share more common metabolic and signaling pathways with the former than microbial cells. Therefore, identifying appropriate targets for antifungal medicines remains a challenge [29]. Since human cells are devoid of a cell wall, whereas fungal cells are endowed with such a wall consisting of polysaccharides (glucan, chitin) and glycoproteins, it has been regarded as “an ideal target” [29]. Although much research has been dedicated to the fungal cell wall as an antifungal target (with good reason), and most currently used antifungals act at the cell wall or plasma membrane levels, other targets have been, until recently, less explored. The flurry of innovation in molecular biology and “omics” sciences in the last two decades has opened the door wide for exploring other potential targets, which could act synergistically with the conventional antifungals and overcome resistant strains. In this paper, we focus on two related signaling pathways that have attracted much attention in the last years: the calcineurin and heat shock proteins pathways. 

## 3. The Calcineurin Pathway

**Figure 1:**
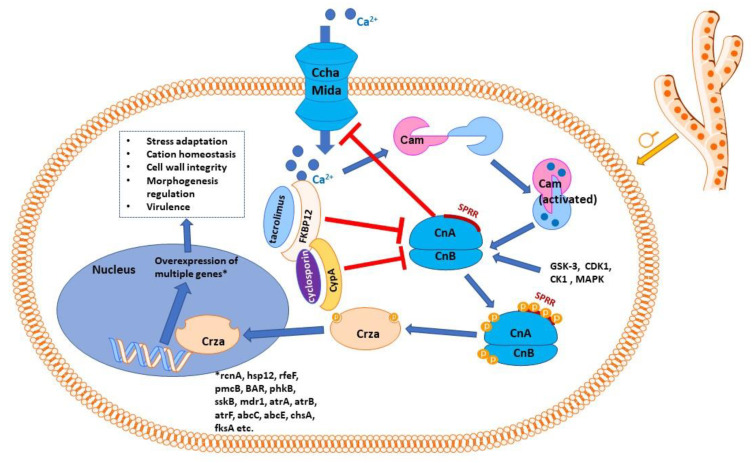
Schematic representation of calcineurin signaling pathway in *Aspergillus* sp. Calmodulin is activated by calcium ions, which penetrate inside the cell through the high-affinity calcium influx system, consisting mainly of the Ccha and MidA calcium channels, the key access route for calcium in the cell when calcium levels are low. CchA, at least, is negatively regulated by calcineurin. The Ca^2+^–calmodulin complex activates calcineurin with its two subunits, catalytic (CnA) and regulatory (CnB). Calcineurin presents a serine–proline-rich region (SPRR); phosphorylating the four serine residues of SPRR, as well as phosphorylation of CnA C-terminus and CnB N-terminus, is necessary for calcineurin to exert its effects. Complexes of immunophilins with immunosuppressant molecules (FK506-binding protein with tacrolimus or cyclophilin A with cyclosporin) inhibit calcineurin. Activated (phosphorylated) calcineurin acts on its effector protein, CrzA (the zinc finger transcription factor Crz1 homolog), dephosphorylating it. Dephosphorylated Crza is translocated into the nucleus, where it activates the transcription of a number of genes, which encode proteins involved in cation homeostasis (rcnA, pmcA-pmcB), other proteins involved in stress response (sskB, hsp12), efflux proteins (mdr1, atrA, atrB, atrF, abcC, abcE), proteins responsible for cell wall integrity (chsA, fksA), proteins involved in regulating morphogenesis and in virulence (BAR, but also pmcA, sskB, etc.). As for many such diagrams, only the most relevant molecules and interactions are shown; in reality, the signaling network is more complex and currently only partially known.

Calcineurin is a serine/threonine phosphatase activated by elevated concentrations of calcium, which connects upstream calcium signaling pathways to downstream protein signaling through changes in phosphorylation states [30]. It has a large number of substrates and binding partners and a wide range of cell functions, orchestrating a diverse array of cell events and processes [31]. Calcineurin is activated by Ca^2+^-calmodulin (Figure 1), apparently the only phosphatase that needs both calcium and calmodulin to exert its enzymatic function [32]. It is interesting in this context to mention that known antifungal azoles inhibit calmodulin in a concentration-dependent fashion, and it has been speculated that this inhibition could contribute to their antifungal effects (besides their assumed primary mechanism of ergosterol biosynthesis inhibition) [33]. 

Calcineurin is a heterodimer protein consisting of two chains: a catalytic subunit (calcineurin A) and a regulatory subunit (calcineurin B) [34]. Alterations in human calcineurin signaling have been implicated in numerous pathologies, from heart diseases [35] to autoimmune pathologies [36], from nociception [37] to new-onset diabetes mellitus following transplantation [38]. In various fungi species, calcineurin has been found to play multiple regulatory roles, e.g., regulating adaptation to stress, growth at higher values of pH and temperature, cation homeostasis, membrane trafficking and reacting to membrane stress, cell wall integrity, regulating morphogenesis, hyphal branching, the appressorium, sclerotial formation, and virulence [39,40,41]. Although calcineurin pathways seem conserved among different fungal taxons, considerable differences have been identified between various genera; therefore, extrapolating from one taxon to another may not be appropriate [39]. 

Lung surfactant protein D (SP-D) has a protective role against various microorganisms, its deficiency being associated with increased susceptibility to bacterial or viral infections [42]. Its binding to *A. fumigatus* hyphae is influenced by the activity of calcineurin. The deletion of the catalytic subunit of the latter (calcineurin A) results in canceling the binding of SP-D to the fungal hyphae [42]. However, the potential clinical application of this finding is still unclear, and more research is necessary to understand its implications. 

Data from different fungal genera, including *Aspergillus* sp., have indicated that calcineurin plays critical roles in the development and virulence of fungi and has an essential effect on the activity of antifungal medicinal products [43]. Mutants of *A. fumigatus* devoid of the catalytic subunit of calcineurin (CnaA) manifested multiple defective phenotypic features, resulting in reduced filamentation and sporulation and markedly attenuated pathogenicity in several animal models [43,44]. Deleting the regulatory unit (*CnaB*) had a similar impact, whereas deletion of both subunits had an additive effect, the double mutants growing slower than each single mutant [39,40]. Furthermore, the hyphal septa were curved, wavy, or otherwise impaired, indicating disorganization of the cell wall related to a marked decrease in its beta-glucan content and a compensatory increase in its chitin content [39,40]. However, this compensatory response of increasing chitin seems diminished in mutants devoid of calcineurin or *Crza* genes (for Crza, see below) [45,46]. *Calcineurin* gene has also been proven to be essential for other *Aspergillus species*, such as *A. nidulans* [47] and *A. oryzae* [48], and calcineurin A from the latter species, which has a functional homology with that from yeast [49]. Conidia of *A. nidulans* with disrupted calcineurin A germinate in a proportion of less than 50%, and conidia with disrupted calmodulin germinate only in a percentage of about 60% [47,50,51]. Growth defects at the hyphae levels observed under calcineurin deletion/suppression are canceled with the deletion of the high-affinity calcium channel CchA, the latter having, as a consequence, a decrease in cytoplasmatic Ca^2+^ concentrations [52].

In *A. niger*, deleting calcineurin catalytic subunit or other proteins from the calcineurin signaling pathway resulted in a reduced biofilm formation, lower hydrophobicity and adhesive abilities of conidia (which are necessary for biofilm formation), perturbation of hyphae cell wall structure and hyphae flocculation (and thus also affecting biofilm formation) [53]. 

Aspergillosis results from inhaling *Aspergillus* conidia (asexual spores) by immunocompromised patients. Once inhaled, the spores will germinate and form filamentous hyphae, whose growth is essential for the occurrence and then maintenance of invasive aspergillosis [51]. Henceforth, arresting or hindering hyphal growth is critical in controlling and preventing the disease, but hyphal growth physiology is currently little understood. The discovery of calcineurin roles in hyphal development has suggested that this signaling pathway is pivotal for controlling invasive aspergillosis, hence the particular interest in its chain links as potential targets for antifungal products [51]. 

In vitro, cyclosporin (a calcineurin inhibitor employed as an immunosuppressant) was shown to significantly decrease colonies of *A. fumigatus* in a dose-dependent manner, tending to confirm the hypothesis that calcineurin inhibition results in antifungal effects [54]. Chromofungin is a short peptide fragment derived from chromogranin A (a peptide secreted by chromaffin cells from the adrenal medulla). It has been claimed that the antifungal effect of chromofungin is related to its inhibition of calcineurin, but this seems a somewhat speculative statement, considering that at the relatively high concentration of 5 μM, calcineurin is only inhibited in a proportion of about 32%. In contrast, complete inhibition could be obtained at 250 μM [55]. 

Structurally diverse inhibitors of calcineurin, such as tacrolimus (FK506), cyclosporin, FK520, and L685,818, have all demonstrated a synergistic effect when associated with caspofungin, as well as with other antifungals, such as itraconazole or cycloheximide [56,57,58]. Such an effect may be attained with concentrations of calcineurin inhibitors usually found in the blood of patients treated for immunosuppression purposes [59]. In vitro, caspofungin and tacrolimus consistently demonstrated antifungal activity against 12 clinical isolates of *A. fumigatus*, but associated with caspofungin, they showed slightly additive effects at best and more likely no interaction (FICI for tacrolimus + caspofungin was 0.85 for isolates from transplant recipients and 1.05 for isolates from non-transplant patients; FICI between 0.5 and 4 are interpreted as indicating no interactions [60]) [61]. Three motifs involved in the interaction between calcineurin and its substrates have been identified and characterized up to now [62]. 

Moreover, it was found that the so-called paradoxical effect of caspofungin seems to be mediated by calcineurin (activated through increased Ca^2+^ concentrations); therefore, its pharmacological inhibition is able to cancel it [46,63]. Tacrolimus demonstrated additive (but not synergistic) effects in vitro on *A. fumigatus* when associated with polyhexamethylene biguanide, amphotericin B, or voriconazole [9]. The association of cyclosporin with voriconazole in vivo resulted in worsened effects of the azole antifungal compared with the monotherapy. Although tacrolimus and cyclosporin act through the same mechanism (inhibiting the calcineurin pathway), they bind to different proteins of the immunophilin family, tacrolimus to FKBP12, whereas cyclosporin to cyclophilin, and it was speculated that this could explain the difference between the effects of the two calcineurin inhibitors [9].

Early on, with the discovery of the importance of the calcineurin signaling pathway in fungi, researchers have been asking the question of how feasible it is to target calcineurin in vivo for antifungal effect, given its role in the mammalian immune system and the fact that there was an extensive clinical experience with calcineurin inhibitors in organ transplantation patients [64]. This was a logical and obvious concern because host immunosuppression brought by calcineurin inhibition could be clinically more relevant than the inhibition of fungal growth by the same mechanism, with the consequence of a lack of effectiveness or even exacerbation of the fungal infection [64]. In humans, the calcineurin–NFAT signaling pathway is strongly involved in myeloid progenitor maintenance, and inhibiting this signaling pathway could hinder the activity of the myeloid cells and increase the sensitivity of immunosuppressed patients to invasive aspergillosis [65]. Moreover, it is known that patients treated with calcineurin inhibitors are also much more susceptible to invasive aspergillosis. More recent research has suggested that activation of calcineurin–NFAT by TLR9 results in impaired recruitment of neutrophils and fungal killing, explaining this increased patient susceptibility to fungal infections [66,67] and cautioning one to the use of calcineurin inhibitors as potential antifungal agents. Calcineurin also orchestrates a lateral transfer of *A. fumigatus* between macrophages in a necrosis-dependent process; inhibiting calcineurin results in escaping the immune system by the fungus [68]. 

Furthermore, in heart transplant recipients, unlike patients treated with other immunosuppressive agents (azathioprine, rabbit antithymocyte globulin), patients receiving cyclosporine had a lower rate of invasive aspergillosis (11% vs. 24%), as well as of viral or bacterial infections and lower mortality [69]. Another study compared the infection and mortality risks in two mini-cohorts of liver transplant recipients, one older and one newer. The former used less tacrolimus and more cyclosporin, the latter less cyclosporin and more tacrolimus. The authors found that the new cohort (with more tacrolimus) had less disseminated aspergillosis than the former [70]. Both of these studies were observational in nature and used historical controls; therefore, their methodological limitations should be taken into account. Some researchers have seen in these studies evidence for a potential clinical benefit of calcineurin inhibitors in preventing or controlling aspergillosis [61]. Others could look at the empty half and remark that a sizeable proportion (11–24%) of patients treated with calcineurin inhibitors still falls victim to invasive aspergillosis; thus, if beneficial as antifungal, they are definitely imperfect. Experimental data from rabbits showed that challenging the animals with the same inoculum administered intratracheally, rabbits with granulocytopenia induced by araC had a 100% mortality, and rabbits receiving cyclosporin A and methylprednisolone had a 100% survival, whereas in the later conidia germinated abundantly, mature hyphae were rare unlike the granulocytopenic animals [71]. Furthermore, in a murine invasive aspergillosis model, animals treated with cyclosporin survived significantly less than animals treated with vehicle only, as well as less than animals treated with other immunosuppressant agents, also inhibitors of calcineurin. Whereas the median survival for mice treated with cyclosporin was three days, for the vehicle it was 6.5 days (*p* = 0.001), for tacrolimus 6.5 days (*p* = 0.03), and for sirolimus 1 and 10 mg/kg 7.5 and 9.5 days (*p* = 0.002 and *p* = 0.001) [72].

## 4. Other Targets from the Calcineurin Pathway

A potential solution to avoid the negative consequences of immune suppression of calcineurin inhibitors while reaping the antifungal benefits would be to use a method of direct pulmonary delivery (for pulmonary aspergillosis) [51]. 

A potentially better option could be to target proteins from the calcineurin signaling pathway that are only present in fungal species but not in mammals. One such target is the zinc finger transcription factor Crz1 homolog (CrzA). This calcineurin effector protein is analogous to the mammalian NFAT (nuclear factor of activated T-cells) [64]. Whereas calmodulin and calcineurin are extensively conserved across fungal and mammalian cells, wide heterogeneity exists for their downstream components, with CrzA and NFAT1 sharing only 15% amino acid sequence identity [73]. Calcineurin triggers the dephosphorylation of CrzA, which is then translocated to the nucleus, where it influences the transcription of a plethora of genes involved in crucial cell processes [73]. Among others, nuclear translocation of CrzA induces overexpression of multiple multidrug transport genes (*mdr1, atrB, atrF, abcE, atrA*, and *abcC*), which could explain resistance to antifungals such as azoles [74,75]. Based on the available evidence, it is also believed that calcineurin (in concert with Hsp90) acts similarly (through CrzA) to increase the expression of enzymes involved in cell wall repair, chitin synthases (chsA-G), and β-glucan synthase (fksA) [29,32] (Figure 1).

Deletion of the *crzA* gene in *A. fumigatus* gives rise to serious defects in conidium production and germination, in the polarized growth of the hyphae and a marked decrease in hyphal growth (a 68% reduction), alterations in the cell wall structure, as well as alterations in hyphal morphology; these alterations were as not as severe as those induced by calcineurin A deletions [64]. Moreover, deletion of the *CrzA* gene in *A. fumigatus* resulted in reduced virulence in a murine model of invasive pulmonary aspergillosis [76]. The fact, however, that calcineurin deletion results in growth defects that are more pronounced than those associated with the deletion of *CrzA* indicates that it is not the only target protein of calcineurin [64,76,77]. In *A. nidulans*, deletion of *crzA* results in sensitivity to high extracellular concentrations of Ca^2+^ (reduced radial growth occurs at 10 mM concentrations and complete inhibition at 50 mM concentrations), but high Mg^2+^ concentrations seem to cancel this sensitivity [78]. Thus, whereas *CrzA* is considered a non-essential gene in normal conditions, it becomes essential in the presence of high Ca2+ concentrations [76]. In *A. parasiticus*, not only is *crzA* important in elevated calcium levels tolerance, but it also performs an essential function in aflatoxin biosynthesis, as deleted *crzA* mutants have a shallow ability (if any) to produce the toxin [79]. In *A. flavus*, it was reported that quercetin upregulates both calcineurin and *crz1/crzA* genes, upregulation that was interpreted as a response to quercetin-induced stress [80]. In *A. niger*, *CrzA* deletion results in lower biofilm formation, decreased adhesion and hydrophobicity of conidia, decreased hyphae flocculation ability, and altered cell wall integrity of the hyphae [53]. In *A. niger*, a transcriptomics analysis suggested that cell wall integrity depends on at least three transcription factors, one of which is crzA [81].

Despite the attractiveness of CrzA as a target, site-directed mutagenesis experiments have indicated that effective suppression of this protein might be challenging to achieve because it has a relatively high number of phosphorylated residues, and an important number of kinases are predicted to be involved in their phosphorylation [73]. 

A serine–proline-rich region (SPRR) located in the calcineurin was identified that is conserved among filamentous fungi and is uniquely specific for this group, whereas it is missing from the human versions of the *calcineurin* gene [82]. Blocking phosphorylation of this region through mutations of the serine residues resulted in substantial defects affecting hyphal growth, and in attenuated virulence, opening a new way of targeting the calcineurin pathway in *Aspergillus* sp. and other fungi without depressing the immune response of the host [82]. Several proline-directed kinases have been identified to date that are capable of phosphorylating the four serine residues from SPRR (GSK-3, CDK1, CK1, and MAP kinase), but several others seem unidentified as yet, and further investigations are necessary to clarify the enzymes involved and how to inhibit them [32]. 

Data generated on *CrzA*-deleted *A. fumigatus* indicated that potentially attractive targets (as implied by impressive mRNA increase or decrease) could be Hsp9-12 heat shock protein Scf1 homolog (Afu1g17370; hsp12 is discussed below, together with other heat shock proteins), rcnA (Afu2g13060, a calcipressin), pmcB (Afu3g10690), rfeF (Afu4g10200), and BAR adaptor protein (Afu3g14230) [83]. CrzA also induces the expression of several members of the high-osmolarity glycerol response (HOG) pathway, including phkB histidine kinase (Afu3g12530) and sskB MAP kinase kinase kinase (Afu1g10940) [84]. 

*rcnA* was initially identified as a cobalt- and nickel-resistant gene in *E. coli*, which encodes an efflux pump [85]. The *rcnA* gene is involved in responding to oxidative and calcium stress in *Aspergillus* species; it regulates calcineurin effects and the expression of calcium transporters genes (pmcA and pmcB) [83]. pmcA and pmcB are calcium transporters (probably located in the plasma membrane or the vacuole, as their precise location has not been confirmed). *pmcA* and *pmcB* null mutants are more sensitive to increased calcium concentrations but more resistant to manganese or cyclosporin; still, only *pmcA* null mutants were avirulent in a mouse model of invasive aspergillosis [86]. *pmcC* is an essential gene in *A. fumigatus*, and its downregulation results in fungal growth inhibition [86], suggesting that this calcium transporter is an alluring target for antifungal development. BAR (Bin/Amphiphysin/Rvs) adapters are versatile proteins that belong to a superfamily of structurally related proteins, among which Bin1 and Bin3 are considered most characteristic and highly conserved, from fungi to primates [87]. Data from *C. albicans* suggest that some BAR domain proteins are engaged in endocytic processes, yeast-to-hyphae transition, as well as in the virulence of this species [88]. 

sskB is a mitogen-activated protein kinase kinase kinase (MAPKKK) of the high-osmolarity glycerol (HOG) signaling pathway, with roles in helping cells cope with osmotic stress [89]. sskB null mutants are more sensitive to calcium and other stress sources (NaCl, paraquat) and are devoid of virulence in a mouse model of invasive lung aspergillosis [84]. 

The family of calcineurin-binding proteins, also known as calcipressins, also widely conserved from fungi to humans, has drawn attention as a potential target within the calcineurin signaling pathway [90]. Among these, calcineurin-binding protein A (CbpA) was investigated in several fungal species. Deleting the *CbpA* gene in *A. fumigatus* resulted in a phenotype with slightly impaired hyphal growth and somewhat weakened virulence [90]. Three domains of CbpA may be phosphorylated: PD-I, PD-II, and PD-III. Whereas mutation of the phosphorylated serine residues in PD-II did not have any impact on the Cbpa function in vivo, mutation of two serine residues subject to phosphorylation (Ser156 and Ser160) in PD-1 results in dwindled hyphal growth and increased susceptibility to oxidative stress [91]. 

Deletion of calcineurin A subunit results in overexpression of four transcription factors: zfpA (a C_2_H_2_ transcription factor encoded by *Afu8g05010*), htfA (a C6 transcription factor encoded by *Afu4g10110*), nosA (encoded by *Afu4g09710*), and ctfA (a C6 transcriptor factor encoded by *Afu1g17460*) [92]. *A fumigatus* strains with deletions of the genes encoding these transcription factors were less susceptible to azoles or echinocandins [92]. This suggests that activating these transcription factors might have an antifungal effect (or at least increase susceptibility to conventional antifungals), but this will have to be confirmed experimentally. Furthermore, several genes involved in translation and mitochondrial processes were downregulated [92]. Still, these might be more difficult to identify and selectively target, considering that translation and mitochondrial processes tend to be widely conserved in the living world. 

The catalytic subunit of calcineurin (calcineurin A) interacts in *A fumigatus* with a nucleoside diphosphate kinase (SwoH). The gene encoding the enzyme (swoH) was demonstrated to be an essential gene. A point mutation in its active site resulted in a curtailed growth and enhanced sensitivity to relatively high temperatures (37 °C) [93]. In an in vivo experiment on honeycomb moth (*Galleria mellonella*), whereas at 30 °C, there was virtually no difference in virulence between the wild-type and the mutated SwoH^V83F^ strains, at 37 °C, the mutated strain caused the killing of only about 20% of the larvae. In contrast, the wild-type (as well as the complemented SwoH^V83F^:SwoH^+^ strains) killed 100% of the larvae [93]. These data suggest that SwoH could be another attractive target from the calcineurin pathway to be explored for antifungal development. 

The calcium channel CchA is negatively regulated by calcineurin, and it was reported that its deletion canceled *A. nidulans* and *A. fumigatus* susceptibility to increased external calcium concentrations [94]. Ccha, together with a second channel (MidA), form together the so-called high-affinity calcium influx system (HACS), which is the main access route for calcium in the cell when calcium levels are low [95]. Both channels were shown to be essential for spore development, formation of hyphal polarity, and cell wall architecture [95]. Deletion of *Ccha* and *MidA* resulted in attenuated virulence for those fungal strains in an invasive pulmonary aspergillosis rodent model [96]. Deletion of *Ccha* or *MidA* was also reported to have similar effects to those of deleting *CrzA* or *CnA*: decreased biofilm formation, diminished hydrophobicity and adhesion of conidia, weakened flocculation ability, and altered cell wall structure of the hyphae [53]. When calcium levels are high, the so-called low-affinity calcium influx system (LACS) is operating, Fig1 (FigA in the case of *Aspergillus*) being its only member known in fungal species; Fig1 null mutants manifest hyphal growth defects and a sizeable decrease in conidial production [97]. Adding extracellular Ca^2+^ in the growth medium could reverse the hyphal growth defects but could not save the sexual or asexual reproduction failures [97].

While CchA and MidA may be the best-known proteins involved in calcium metabolism and signaling in *Aspergillus*, they are not the only ones involved, the process being more complex and only partially understood. YvcA is a vacuole calcium channel involved in calcium metabolism and homeostasis. *YvcA* null mutants were avirulent in a mouse model of invasive pulmonary aspergillosis [96]. VcxA is a vacuolar Ca^2+^/H^+^ exchanger whose expression seems to be increased by calcineurin and CrzA in *Aspergillus*, unlike other fungal species where it is, contrarily, repressed [98]. PmrA is a Ca^2+^/Mn^2+^ P-type ATPase located in dictyosomes and has a role in maintaining Ca^2+^ homeostasis and providing Mn^2+^ necessary for protein glycosylation in the Golgi apparatus [99]. Although *pmrA* null mutants manifested certain growth defects and increased susceptibility to antifungal substances (together with an increased expression in calcineurin signaling), they did not differ in their pathogenicity, as demonstrated in a murine model [99]; therefore, its interest as an antifungal target could be reduced. AkrA is a palmitoyl transferase (a homolog to *S. cerevisiae* palmitoyl transferase ScAkr1p) located in the Golgi apparatus, whose function in low extracellular calcium conditions is similar to HACS, i.e., it is part of the calcium-signaling machinery [77]. Several proteins, including a Pmc1 homolog (pmcA-pmcC mentioned above, are Pmc1 homologs) and three putative CrzA-dependent proteins, seem to be palmitoylated by AkrA [77]. *AkrA* null mutants manifest less severe growth defects than calcineurin mutants. Still, double null mutants (*Akra* and *CnaA*) display even more severe defects and less conidiation, suggesting that Akra and calcineurin perform different functions and act through different pathways [77]. Calnexin is a molecular chaperone widely conserved throughout eukaryotes, localized in the endoplasmic reticulum, also performing functions similar to HACS. As for the AkrA case, double null mutants show more severe growth defects than single gene mutants [100]. Finally, McuA is a uniporter responsible for mitochondrial uptake of Ca^2+^, and its deletion results in increased susceptibility of *A. fumigatus* to azoles [101]. It is not clear to what extent McuA interacts with the calcineurin pathways.

Whereas Kin1 protein kinase, which is a member of the PAR-1/MARK/MELK protein family, is necessary for growth and pathogenesis in several fungal species, it was shown to be of minimal relevance to *A. fumigatus* [102] and thus of little interest, if any, as a potential antifungal target in this species. 

## 5. Immunophilins

Cyclophilins and FK506-binding proteins (FKBP) are two protein families, remarkably conserved, with a high affinity for immunosuppressive drugs, jointly known as immunophilins [103]. FKBP12 (12-kDa FK506 binding protein) is a widespread cytosolic protein and the target of tacrolimus (FK506), whose binding by FKBP12 is essential for the inhibition of human calcineurin by this immunosuppressant drug [104]. Four orthologous genes of FKBP12 were identified, among which the first (fkbp12-1) is the primary mediator of tacrolimus binding to calcineurin. The fourth seems to play some role in basal fungal growth and the so-called paradoxical effect associated with caspofungin [105]. 

L-685,818 is a competitive antagonist of tacrolimus that binds to FKBP12, but it has no calcineurin inhibitory effects and is devoid of immunosuppressive activity [106]. It is claimed that it manifested antifungal activity against *Cryptococcus neoformans* (slightly less than tacrolimus, but with 10^5^-fold less immunosuppressive effects) [107]; however, it displayed no antifungal effects against *A. fumigatus* [56,57]. More recently, M. Nambu et al. (2017) reported synthesizing a number of tacrolimus analogs that inhibit FKBP12 and antagonize tacrolimus in human cells but not in fungal cells. In combination with tacrolimus, two of these compounds caused a proliferation reduction of *A. fumigatus* of 22 and 24% compared with the negative controls (tacrolimus would exert its antifungal effects, whereas its antagonists would prevent immunosuppression in the human body) [108]. Whether a 20–25% proliferation is sufficient to be of clinical significance remains to be seen. 

In *A. niger*, the *cyclophilin A* (*cypA*) genes encode a peptidyl prolyl cis-trans isomerase from the cyclophilin family. It was found that overexpression of *cypA* in this species enhances its susceptibility to the antifungal effects of cyclosporin A, suggesting that the cyclophilin enzyme encoded by *cypA* is the target of the immunosuppressor agent [109]. Heat shock tends to slightly increase its expression and thus the susceptibility to cyclosporin [109].

## 6. Heat Shock Protein 90

The heat shock protein 90 (Hsp90) was first identified at the end of the 1970s from *Drosophila melanogaster*. When the insect is grown at about 25 °C and then exposed to high temperatures (e.g., 37 °C), specific genes are activated and expressed, and their expression products are the heat-shock proteins [110]. It was later found that these proteins are highly conserved throughout all life kingdoms (with the notable exception of archaebacteria) [111]. In 1982, such a protein was isolated from yeast and had a molecular weight of 90,000 Da, being therefore abbreviated to Hsp90 [112]. Due to their high level of conservation, targeting Hsp90 for antifungal effects is challenging. A theoretical way to avoid an unacceptable safety profile because of its high similarity with human Hsp90 would be to identify specific regions vital for fungal virulence or resistance against antifungals which are, at the same time, sufficiently different from the human homologs to avoid cross-reactivity [26]. We now know that Hsp90 is a ubiquitous and abounding chaperone (a protein helping other proteins to adopt an appropriate folding). It is involved in a wide range of cell processes and signaling networks, from survival to the control of the cell cycle, from responding to cell stress to maintaining cell homeostasis [113]. In yeasts, Hsp90 has been shown to control at least 10% of the whole proteome through direct or indirect mechanisms and to play a decisive role in the resistance to azoles and echinocandins [29]. 

**Figure 2:**
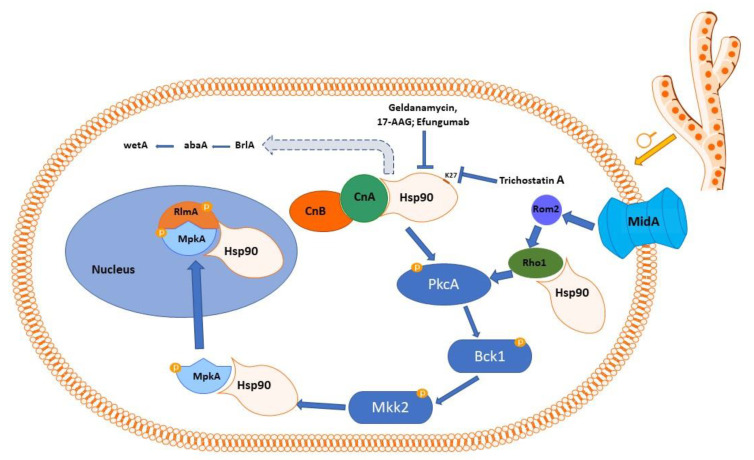
Schematic representation of Hsp90 signaling pathway in *Aspergillus* sp. Calcineurin is chaperoned by Hsp90, which stabilizes it. There seems to be a number of physical interactions between calcineurin and Hsp90, but our understanding of these interactions is currently limited (more evidence is available for *Candida* and less for *Aspergillus*). Protein kinase C (pkcA) is an Hsp90 client (there is also evidence of at least genetic cross-talk between CnA and pkcA). Heat shock response is initiated by the calcium channel MidA, which acts as a stress sensor. The stress signals are integrated by Rom2, which is regarded as the first upstream activator of the cell wall integrity pathway and is a guanine nucleotide exchange factor (GEF). Rom2 activates the Rho1 GTPase, which in turn activates pkcA (Rho1 is also an Hsp90 client) [119,120]. The latter triggers activation in cascade o Bck1 (bypass of protein kinase C), Mkk2 (mitogen-activated kinase kinase), and Mpka (a MAPK, client of Hsp90, known to regulate the expressions of *α-1,3-glucan synthase* genes). Mpka could also be activated by Wsc 1,2,3 proteins, which are general stress sensors at cell wall levels in fungi, although direct evidence in *Aspergillus* seems not to be available yet [121]. MpkA is translocated to the nucleus, where it activates, by phosphorylation, the RlmA transcription factor, whose activity is necessary for the transcription of genes encoding enzymes responsible for cell wall formation and which is also an Hsp90 partner [119]. Furthermore, Hsp90 is somehow involved in activating the expression of *brlA* (possibly also of *abaA* and *wetA*). In its turn, BrlA regulates other genes involved in conidiation, among which the most important seem to be *abaA* and *wetA*.

Inter alia, calcineurin is chaperoned by Hsp90; a relatively large body of evidence in this sense has been generated in *Candida* sp. and, to a lesser extent, in *A. fumigatus* [114]. Hsp90 stabilizes calcineurin (Figure 2), and the latter, as discussed above, orchestrates a number of reactions that facilitate the fungus survival under the challenges of antifungal agents (or other stress factors for the fungal cells); inhibiting the chaperone results in canceling these responses, with a sizeable increase in sensitivity to antifungal agents [114]. 

Although these basic facts would suggest Hsp90 as an attractive target for antifungal development, specifically in the case of *A. fumigatus*, the available evidence is not overwhelmingly positive. In vitro experiments showed that 10-fold reductions of Hsp90 expression are insufficient to have a relevant impact on the in vitro growth of the species [115]. Genetically repressing Hsp90, however, resulted in decreased virulence in a mouse model of invasive aspergillosis; the replacement of its natural promoter with two artificial promoters resulted in increased susceptibility of *A. fumigatus* to caspofungin and a canceling of the so-called paradoxical effect (which describes the observed resistance against caspofungin at high concentrations) [115,116]. Chemical inhibition of Hsp90 using geldanamycin and two of its derivatives (17-AAG and 17-DMAG) had a similar effect of increasing *A. fumigatus* sensitivity to caspofungin [116]. A chemical inhibitor of Hsp90 (radicicol) sharply decreased *A. terreus* resistance to caspofungin. Still, neither radicicol nor geldanamycin had any effect on the basal resistance of *A. terreus* to fluconazole or voriconazole [117]. Chemical inhibition of Hsp90 with geldanamycin or its derivatives, and its genetic repression, were also devoid of any synergistic effect with voriconazole or amphotericin B on *A. fumigatus* [116]. However, G. Blum et al. (2013) claimed a substantial potentiating effect for the chemical inhibition; the association of the antifungal with geldanamycin or 17-AAG (5–20 μM) decreased the MCI of resistant strains from >32 to less than 1 mg/L [118]. 

Assuming there was a benefit in inhibiting Hsp90 (e.g., by potentiating other antifungals, such as caspofungin), the currently used pharmacological inhibitors seem too toxic to entertain hopes for clinical development. Geldanamycin is a natural metabolite isolated initially from *Streptomyces hygroscopicus* and belongs to the group of benzoquinone ansamycins [122]. Although it has exhibited interesting antitumor, anti-angiogenic and anti-metastatic properties in non-clinical experiments, it has poor chances of clinical development because of its hepatotoxicity, inferior oral bioavailability, and low water solubility [29,123]. As a single agent, it has little activity against *Aspergillus* species (MEC_50_ higher than 16 mg/L) [124]. 17AAG (also known as NSC 330507, KOS 953, and Tanespimycin) has lower toxicity, better stability and has been evaluated in various clinical trials [123]. However, in a murine experiment, although the animals receiving amphotericine B plus 17-AAG (20 mg) demonstrated a fall in fungal load, they had inferior survival, the authors of the experiment speculating that the toxicity of the Hsp90 blockers was too high to be used in vivo for the treatment of aspergillosis [118]. These toxic outcomes were observed despite tanespimycin’s lower toxicity than geldanamycin [118]. Similar claims were made earlier by a different group of researchers for Hsp90 inhibitors, without showing the data: “Initial experiments in mice indicated that inhibition of host Hsp90 is not well tolerated in the context of an acute fungal infection (data not shown)” [27]. It is speculated that their inappropriate safety profile is related to their lack of specificity for fungal cells and cross-reactivity against human targets [26]. It can be concluded, therefore, that inhibiting Hsp90 to potentiate echinocandins or other antifungals does not seem to be an opportune approach in light of the available data. More potent and less toxic Hsp90 inhibitors could still offer an opportunity for such associations, but currently, this is only a theoretical possibility.

Monoclonal antibodies against Hsp90 have also been investigated with initial high hopes. Efungumab (trade name Mycograb) was such an antibody developed by NeuTec Pharma, not for treating aspergillosis, but for candidiasis, to be used in association with amphotericin B. The company attempted to obtain a European Union-wide marketing authorization through the European Medicines Agency. Still, the application was rejected because the CHMP concluded by consensus that the risk–benefit balance for the product was negative (after a re-examination request, it reached the same conclusion again) [125]. The main issue that resulted in a refusal of the marketing authorization, however, was rather related to quality issues (the antibody tended to form aggregates, which was not a problem per se, but CHMP required evidence of proper characterization and consistency of manufacturing; the presence of a high level of host cell proteins was a second major quality issue) and to some extent to safety. However, in the re-examination procedure, CHMP agreed that the safety aspects could have been manageable in clinical practice [125]. The utility of such an antibody as an antifungal remains uncertain. A small (*n* = 117 subjects in the ITT population), double-blind, randomized clinical trial reported a large benefit for the combination of efungumab + amphotericin B against placebo + amphotericin B [126]. Because of the quality issues mentioned above, the company developed another variant of the monoclonal antibody (Mycograb C28Y variant, the cysteine at position 28 being replaced by tyrosine); in an in vivo murine model, however, this new variant offered no benefit in association with amphotericin B against amphotericin B alone [127]. It is not clear why this difference was observed between the two antibody variants, but it seems unlikely that a similar antibody will be soon assessed again for its antifungal potential. 

The K27 lysine position from Hsp90 seems to be essential for *A. fumigatus* virulence: its experimental mutation to alanine (a mutation considered to mimic acetylation) was enough to impair protein functioning and induce hypersensitivity to geldanamycin, caspofungin, and voriconazole, whereas other stress responses mediated by Hsp90 remained apparently unaffected [26]. Complete *K27* deletion had similar effects of increasing sensitivity to geldanamycin, caspofungin, and voriconazole, whereas a *K27* mutation to arginine (mimicking a deacetylation state) had little effect. This would suggest that acetylation of K27 should have antifungal effects, whereas its deacetylation should be required for normal Hsp90 functioning in *A. fumigatus* [26]. Trichostatin A, a lysine deacetylase inhibitor that can favor the acetylation of Hsp90 in eukaryotic cells, induced conidiation and growth defects similar to those caused by *K27* alanine mutations [26].

## 7. Other Targets from the Hsp90 Pathway

It has recently been reported that Hsp90 interacts with the cell wall integrity pathway, and at least three components of the latter (PkcA, MpkA, and RlmA) are clients of the former [119]. Moreover, a *PkcA^G579R^* mutation (glycine to arginine mutation at position 579) suppresses this interaction and the rapid activation of MpkA as a response to Hsp90 [119]. PkcA (protein kinase C, *Aspergillus*, equivalent to pkc1 in yeast) seems an essential protein in *Aspergillus nidulans*, as its deletion is lethal; it performs multiple cell functions, from cell wall integrity to conidiation, germination, and secondary metabolism [128]. MpkA is a MAPK enzyme that regulates the expression of two α-1,3-glucan synthase genes (*agsA* and *agsB*) in *A. nidulans* [129,130]. Its deletion in this species results in impaired conidial germination and defective polarized growth, with swellings at the hyphae tips and atypical branches [131]. Sequence similarity among the Mpka from *A. nidulans* and *A. fumigatus* suggests that similar roles are to be expected for the enzyme in the latter species [132]. Rlma is a transcription factor involved in cell wall integrity, acting downstream of PkcA and MpkA, and whose activity is necessary for the transcription of genes encoding enzymes responsible for cell wall formation [133]. In a murine model of invasive pulmonary aspergillosis, *Rlma* null mutants of *A. fumigatus* manifested weakened virulence compared to the regular strains [133], indicating it as a potential target for antifungal activity. The deletion of *Rlma* in *A. niger* resulted in increased sensitivity to cell wall stressors; it triggers an increase in chitin synthesis, as well as increased expression of an α-1,3-glucan synthase (increased transcription of the *agsA* gene) [134] (Figure 2). 

Hsp90 inhibition was reported to associate with a noteworthy reduction in the expression of genes known to be involved in conidiation: *brlA*, *abaA*, and *wetA* [116] (Figure 2). 

BrlA is a transcription factor that has been described as “a master regulator of conidiation” [135]. *A. fumigatus* brlA-deficient mutants are entirely devoid of their ability to form conidiophores (no conidiation) [136], have hyphal lengths augmented to abnormal sizes, have a “bristle” aspect, and are affected by extensive dysregulation of genes involved in spore germination, hyphal growth, and virulence [135,137,138]. BrlA has been discovered to be not only a “master regulator of conidiation” but also a “master regulator of secondary metabolism and other cellular processes” [138], a finding also reported for other *Aspergillus* species, such as *A. clavatus* Desm. [139]. It is somewhat surprising that BrlA overexpression was associated with diminished virulence of *A. fumigatus* in two distinct animal infection models, one in *Galleria mellonella* (Linnaeus) (moth) and one in mouse [135]. Overexpression of *BrlA* results in the termination of hyphal development, but also in the formation of viable spores, which seems to be a survival mechanism that might or might not be exploited for therapeutic purposes [140]. Moreover, it was reported that micafungin (an antifungal product belonging to the echinocandins group) induces an increased expression of *BrlA*, assumed to be a type of stress response to cell wall perturbation triggered by the echinocandin derivative [141]. Wickerhamomyces anomalus WRL-076, a yeast proposed as a means of *A. flavus* biocontrol, when co-cultured with the latter, triggers a reduction in conidia and biomass of *A. flavus*, as well as a downregulation of (inter alia) BrlA [142], suggesting that inhibiting BrlA could be a successful antifungal approach. 

In turn, *BrlA* regulates other genes involved in conidiation, among which the most important seem to be *abaA, wetA, rodA*, and *yA* [89]. *BrlA* is expressed early in the conidiation process, and canceling its expression also results in aborting the expression of those downstream genes [89]. 

AbaA is a leading regulator of phialides (conidiogenous cells) differentiation and functioning, its deletion resulting in conidiophores devoid of spores [143]. AbaA consecutively activates WetA in the late phase of conidia development, its absence resulting in spores with defective cell walls, lacking trehalose, with short viability and high sensitivity to stress [143]. *rodA* is a gene that encodes a small hydrophobic polypeptide known as hydrophobin, which is responsible for the formation of the conidia rodlet layer (rodlets are fascicles of protein microfibrils knitted together in the exterior of the conidia cell wall); its null mutants are hydrophilic and have reduced dispersion abilities, and there is speculation that by the absence of the hydrophobin and rodlets, they could be less resistant to phagocytic cells [144]. The *yA* gene encodes for a p-diphenol oxidase (a multi-copper oxidase, laccase), which converts a yellow pigment precursor to a green pigment, responsible for the green-bluish color specific for mature conidia [145]. 

## 8. Other Hsp Proteins

Hsp40 and Hsp70 are two other protein chaperones whose functions consist of recruiting and transferring client proteins to Hsp90, which will then activate them [146]. Hsp40 (DnaJ) usually acts as a co-chaperone for Hsp70, regulating Hsp70 ATPase activity and polypeptide binding to the latter, preventing premature folding [146]. Although the work on discovering Hsp70 inhibitors has spread over more than three decades, success has been challenging to achieve. Currently, only about 15 such inhibitors are known, classified into six groups based on their binding site on Hsp70 [147]. However, they have been developed with the human hsp70 in mind for their potential antitumor effects, not antifungal effects. One such inhibitor, pifithrin-μ (PFT-µ), was tested in vitro against *A. fumigatus* and other fungal species but was found to be inactive against the broad majority of the species tested (MEC_50_ > 16 mg/L) [124]. Other Hsp proteins, for instance, Hsp60, could also be relevant as antifungal targets, although the experimental evidence for their relevance in *Aspergillus* species is currently very limited [148].

Hsp12 is one of several small heat shock proteins, which often act as holdases, i.e., they have a high affinity for denatured proteins, whom they immobilize until Hsp70 and its co-chaperones reactivate them in an ATP-dependent fashion [149,150]. Data from other fungal species (particularly *S. cerevisiae*) indicate that Hsp12 is involved in response to a variety of stress sources, in modulating the physical properties of the cell wall, as well as in resistance to certain antifungals, for instance, amphotericin B [150]. As mentioned above, data from CrzA null mutants of *A. fumigatus* indicated that the Hsp12 Scf1 homolog (Afu1g17370) could be a target for antifungal development. 

## 9. Conclusions

The last two to three decades have witnessed considerable knowledge gained with respect to *A. fumigatus* molecular biology and, in particular, with respect to the calcineurin–Hsp90 pathways. Although calcineurin has stirred much interest as a potential antifungal target, its immunosuppressive side effects make it less attractive than it would seem at first sight. Direct pulmonary delivery is a potential solution to avoid the negative consequences of immune suppression of calcineurin inhibitors while still enjoying the antifungal benefits of this mechanism, but this is yet to be explored. The situation for Hsp90 as an antifungal species for *Aspergillus* is also not too different. Other targets from the calcineurin–Hsp90 pathway open the door to promising success. Still, their chances are difficult to assess at present because few, if any, small molecules or monoclonal antibodies have been developed and tested specifically on these potential targets. Rather, these proteins/genes await validation as antifungal targets. Protein–kinases phosphorylating the four SPRR serine residues, CrzA, rcnA, pmcA-pmcC (particularly pmcC), rfeF, BAR adapter protein(s), the phkB histidine kinase, sskB MAP kinase kinase kinase, zfpA, htfA, ctfA, SwoH (nucleoside diphosphate kinase), CchA, MidA, FKBP12, the K27 lysine position from Hsp90, PkcA, MpkA, RlmA, brlA, abaA, wetA, and other heat shock proteins (Hsp70, Hsp40, Hsp12) currently hold promise and are worth further investigation to target for antifungal effects. Whereas impairing the functionality of some of these targets with drug inhibitors/antagonists could not be sufficient to obtain a clinically relevant response, simultaneously targeting two or more of these proteins might hold the key to success (either by multi-target single molecules, as mentioned for azoles, or by a combination of single-target molecules, as already proven in the case of tuberculosis or AIDS [151]). Although no inhibitor/antagonist is currently known for most of these target proteins, the progress made in computational approaches to drug discovery in the last two decades allows for hope of a more rapid development of such molecules than in the past.

## Data Availability

Not applicable.
